# Tracking electrons at the space-time limit

**DOI:** 10.1038/s41566-026-01932-0

**Published:** 2026-07-03

**Authors:** S. Maier, R. Spachtholz, K. Glöckl, C. M. Bustamante, S. Lingl, M. Maczejka, J. Schön, A. Riedel, K. Richter, F. J. Giessibl, F. P. Bonafé, M. A. Huber, A. Rubio, J. Repp, R. Huber

**Affiliations:** 1 Department of Physics, Regensburg Center for Ultrafast Nanoscopy, and Halle-Berlin-Regensburg Cluster of Excellence CCE, University of Regensburg, Regensburg, Germany; 2 Max Planck Institute for the Structure and Dynamics of Matter, Hamburg, Germany; 3 Initiative for Computational Catalysis, Flatiron Institute, New York, NY USA

**Keywords:** Ultrafast photonics, Quantum physics, Microscopy

## Abstract

The dynamics of an electronic wavefunction often have non-trivial consequences on its spatial distribution, for example, during tunnelling or chemical bond formation. Yet, revealing spatio-temporal coupling requires ultrafast videography at the intrinsic size of electronic wavefunctions, at the so-called space-time limit. Here we experimentally access the intrinsic quantum motion of individual electrons at the space-time limit while they are tunnelling through an energy barrier, using atomic-scale lightwave-driven scanning tunnelling microscopy with attosecond time resolution. While modulating the tunnelling barrier with two time-delayed near-infrared pulses forming phase-controlled single-cycle waveforms, isolated electron tunnelling transients shorter than 1 fs are identified. The measured spatial extension depends on the interplay of multi-photon and field-driven dynamics, as confirmed by full quantum simulations. We experimentally localize the attosecond-confined tunnelling wave packet on the angstrom scale and use it to map a single copper adatom on a silver surface. This fusion of attosecond science with atomic-scale scanning tunnelling microscopy makes it possible to study wavefunction dynamics inside atoms, molecules and solids.

## Abstract (web summary)

Atomic-scale lightwave-driven scanning tunnelling microscopy with attosecond time resolution observes individual electrons while they are tunnelling through an energy barrier.

## Main

**Fig. 1:**
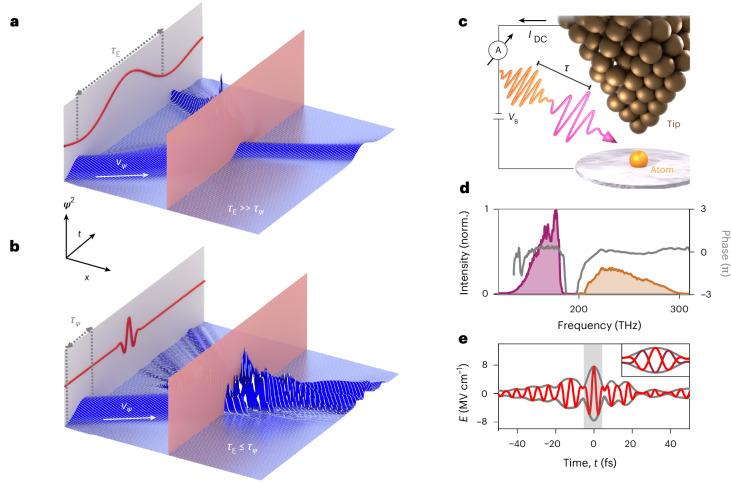
Attosecond lightwave STM. **a**, Quantum mechanical wave packet ψ(x,t) scattering at a barrier (red transparent sheet) in the presence of a time-dependent electric field $E\left(t\right)$ (red wave). The field E(t) oscillates on a timescale ${\tau }_{E}$ larger than the encounter time ${\tau }_{\psi }$ of ψ at the barrier. The spatio-temporal structure of the transmitted ψ is therefore almost unaffected. **b**, Analogous situation, but for ${\tau }_{E}\le \,{\tau }_{\psi }$. The temporally confined interaction imprints a non-trivial spatio-temporal structure on the transmitted ψ. **c**, Schematic set-up: two spectrally distinct NIR pulses with variable delay time τ are focused onto the sharp metal tip of a scanning tunnelling microscope. The bias voltage ${V}_{{\rm{B}}}$ is applied to the sample with respect to the tip. The CEP-dependent part ${I}_{{\rm{CEO}}}$ of the total tunnelling current ${I}_{{\rm{DC}}}$ is extracted. **d**, The normalized intensity spectra of the two incident NIR pulses show no overlap, excluding spectral interference between them. The spectral phase (grey) is flat across most portions of the spectrum. **e**, Resulting single-cycle electric field waveform (FWHM = 5.2 fs) at τ=0 fs and ${\varphi }_{{\rm{CE}}}$ = 0 featuring a pronounced field asymmetry with a peak electric field strength of up to 7.6 MV cm^−1^ in the far field. Grey curve: field envelope. Inset: electric field transients for ${\varphi }_{{\rm{CE}}}=0$ (red) and ${\varphi }_{{\rm{CE}}}=\uppi \,$ (dark red) of the grey-shaded region around *t* = 0 fs. Source data

In quantum mechanics, a spatially localized electron (uncertainty, Δx) is described by a wave packet containing a broad momentum distribution of width Δp bounded by Heisenberg’s uncertainty product^1^ $\Delta x\times \Delta p\gtrsim \hslash /2$. It is, thus, impossible to localize an electron simultaneously in real and momentum space with arbitrary precision. By contrast, there is no uncertainty product between time and space. Hence, one might assume that preparing the temporal structure of a wavefunction (width, Δt) leaves its spatial extent (Δx) unaffected. Yet Δt and Δx are often coupled in non-trivial ways when electrons are subject to ultrafast changes^2–8^. For the prototypical situation of an electron tunnelling through an energy barrier, the Kramers–Henneberger theorem^2,3^ shows that a time-dependent tilt of the barrier—as imposed by phonons^9,10^ or external fields^11–19^—has the same effect as a dynamic spatial displacement. Hence, the spatial shape of the wavefunction may also be dramatically affected depending on the modulation frequency (Fig. 1a,b).

Dynamical reshaping of real-space wavefunctions is believed to play a crucial role also in the making and breaking of chemical bonds^8^, where ionic motion or electromagnetic fields change the energy landscape. In prospective petahertz electronics, ultrafast acceleration of Bloch electrons through a crystalline solid can lead to interband quantum interferences expected to reshape the real-space wavefunctions^20,21^. Inside atoms, electrons approaching the nucleus can be accelerated so strongly that the effective blur of the real-space wavefunction caused by Zitterbewegung changes their fine structure^22^. Yet a direct observation of this class of non-trivial spatio-temporal coupling is extremely challenging as it requires ultrafast atomic-scale videography resolving the space-time volume of the wavefunction, often also referred to as the space-time limit^23^. Ultrafast microscopy has tracked elementary dynamics on ever shorter scales^5,6,20,23–26^. Lightwave-driven scanning tunnelling microscopy (STM) in the terahertz (THz) spectral range has enabled slow-motion imaging of molecular orbitals and defect states^9–19^ with a resolution of ~100 fs (refs. ^12,13^) or even 30 fs (ref. ^27^). On these timescales, electrons follow the nuclear motion instantaneously according to the Born–Oppenheimer approximation, and dynamical reshaping can be neglected. A direct observation of single-electron motion at their intrinsic timescale requires wave packet control within 1 fs or faster^28–31^.

Here we develop sub-femtosecond lightwave-driven STM to sculpt and trace the space-time volume of an attosecond electronic wavefunction. Using a two-colour pump–probe scheme with phase-controlled near-infrared (NIR) single-cycle fields, we directly reveal optically driven wavefunction dynamics faster than a cycle of light. Intriguingly, the volume of single tunnelling electrons depends sensitively on the interplay of multi-photon and field-driven dynamics, as confirmed by full quantum dynamical simulations. We experimentally identify conditions for maximally compact electron wave packets, which allow us to combine attosecond and atomic-scale resolution in real-space microscopy, for the first time.

## Preparation of attosecond wave packets

To prepare compact electronic wave packets, we extend the concept of lightwave-driven STM to the intrinsic timescale of electronic motion illustrated in Fig. 1a,b. Single-cycle light pulses are focused onto the tunnelling junction of an STM (Fig. 1c). Previously, THz biasing has driven electrons in the strong-field regime (characterized by a Keldysh parameter $\kappa \ll 1$; Methods, ref. ^32^) to tunnel across the tip–sample junction during the field crest of the most intense oscillation half-cycle^9,10,12,13,17,18^. Upscaling the carrier frequency by two orders of magnitude from 1 THz to an NIR frequency of ~190 THz may naively be expected to downscale the time window for tunnelling in inverse proportion from ~100 fs to 0.5 fs. Sub-femtosecond charge transfer has indeed been explored in nanostructures^33–35^ or attosecond field emission from metallic nanotips^36–40^. Yet the electrons may no longer react instantly to the field owing to, for example, photon-assisted tunnelling processes^41^.

**Extended Data Fig. 1:**
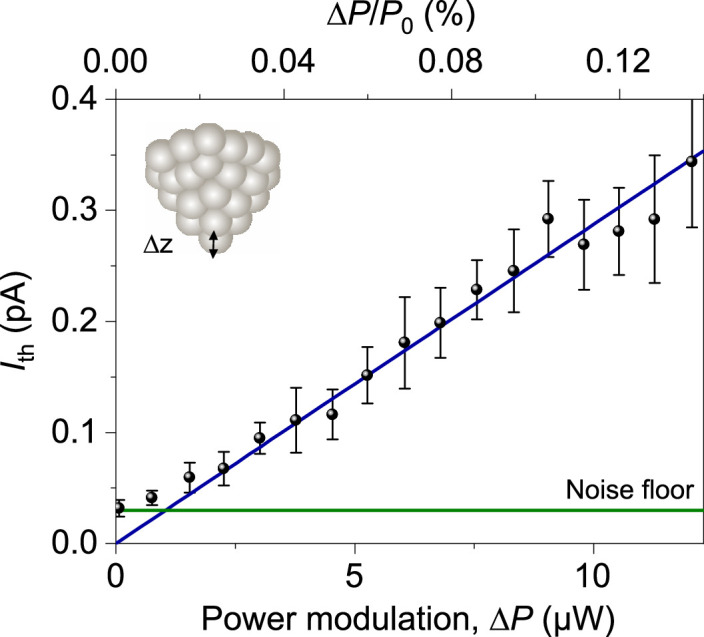
Effect of laser power modulations on DC-current. Even microwatt-level modulations ΔP of the incident laser power *P*_0_ = 8.5 mW (106 pJ) (relative modulation depth $\Delta P/{P}_{0} \sim$10^−4^) lead to appreciable modulations ${I}_{{\rm{th}}}$ of the DC current, owing to thermal effects (current set point, 100 pA at 200 mV bias). The offset is given by the noise floor ( ~ 30 fA, green line). ${I}_{{\rm{th}}}$ increases linearly with the power modulation (see linear fit, blue curve; slope 0.029 pA/µW). Data are presented as mean values, and the error bars correspond to the standard deviation derived from ten consecutive measurements. Source data

This crossover regime, which is characterized by $\kappa \approx 1$ (Methods), can lead to intriguing correlations between the temporal and the spatial structure of the electronic wave packet. We explore these dynamics by focusing NIR pulses onto the tip–sample junction of a low-temperature STM and recording the light-induced tunnelling current (Fig. 1c). Importantly, the NIR light constitutes a critical thermal load on the tip–sample junction. Slight variations of the pulse energy introduce modifications of the tunnelling current that can exceed the coveted lightwave-driven current by many orders of magnitude (Methods and Extended Data Fig. 1). Thermal artefacts can be excluded by keeping the relative fluctuations of the laser power below 10^−4^ (Methods), precluding beam chopping for signal retrieval^42^.

**Extended Data Fig. 2:**
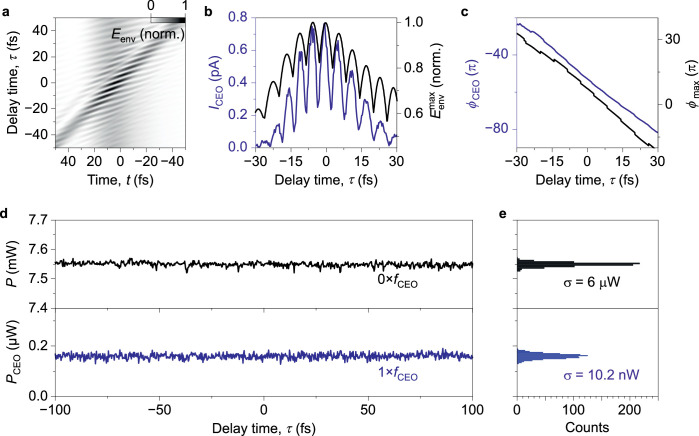
Correlating the current response *I*_CEO_(${\boldsymbol{\tau}}$) with the field maximum ${\boldsymbol{E}}_{{\mathbf{env}}}^{\mathbf{max}}({\boldsymbol{\tau}})$ of the single-cycle waveforms. **a**, Intensity map of the electric-field envelope *E*_env_ as a function of time *t* and delay time τ. The map represents the envelope of the superposition of the two ultrashort laser pulses reconstructed from their optical spectrum. As τ is varied, the field envelope alternates between single- and few-cycle waveforms. **b**, Comparison of ${I}_{{\rm{CEO}}}\left(\tau \right)$ and the maximum field envelope ${E}_{{\rm{env}}}^{\max }$ as a function of the delay time τ. Both quantities exhibit pronounced oscillatory behaviour, with ${I}_{{\rm{CEO}}}\left(\tau \right)$ directly following the oscillations of ${E}_{{\rm{env}}}^{\max }\left(\tau \right)$. **c**, Carrier phase at the field maximum, $\it{\phi }_{\max }$, and corresponding measured phase as a function of τ. In both cases, a predominantly linear dependence on the delay time is observed, demonstrating quantitative agreement between the extracted phase and the experimentally measured signal. **d**, Laser power as a function of the pump-probe delay time τ (black). The average power *P* remains constant at 7.55 mW and exhibits only small, τ -independent fluctuations. The demodulated power at *f*_CEO_ is likewise independent of τ. **e**, Histograms of the data shown in **d**. The standard deviation of the average laser power is 6 µW (0.18% of the mean power, grey). The histogram of the demodulated signal at *f*_CEO_ (light blue) shows an average modulation of 160 nW with a standard deviation of 10.2 nW. Source data

We introduce a novel modulation scheme to separate the lightwave-driven tunnelling currents from trivial background effects: using a newly developed Er:fibre laser system (Methods), we rapidly modulate the carrier-envelope phase (CEP), ${\varphi }_{{\rm{CE}}}$, of the optical waveforms. Our design based on an acousto-optic phase shifter followed by a fibre amplifier and a highly nonlinear optical fibre eliminates power fluctuations and keeps both the beam pointing and the spectral phase of the pulses stable (Methods). The above-octave-spanning spectral intensity and phase after pulse compression (Fig. 1d) define two well-separated pulses centred at *ν*_c_ = 164 THz (purple) and 249 THz (orange), whose mutual delay τ can be tuned with attosecond precision. A large-aperture mirror focuses the pulses onto the STM junction. Because the two NIR pulses do not spectrally overlap, they cannot interfere, excluding average power modulations as a function of τ (Extended Data Fig. 2). This constant-power CEP-sensitive detection scheme enables pump–probe type experiments with subcycle resolution as shown below.

**Extended Data Fig. 3:**
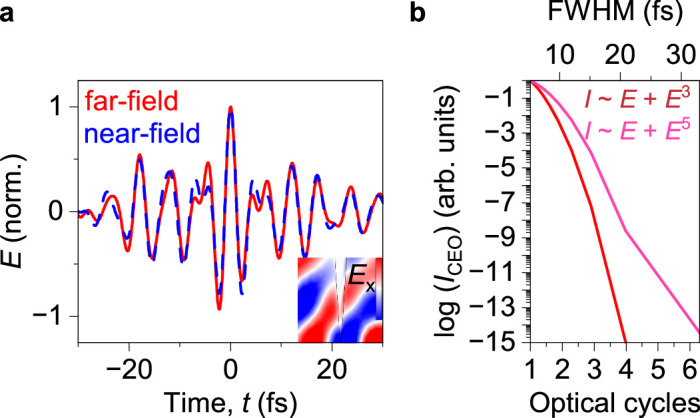
Comparison of far-field and near-field transients obtained from Finite-Difference Frequency-Domain (FDFD) simulations and implications for the time resolution of single-cycle versus multi-cycle pulses. **a**, Experimental far-field transient (red) and retrieved near-field transient (blue). The near-field closely follows the far-field waveform and exhibits a dominant half-cycle that drives attosecond, atomic-scale tunnelling currents. **b**, CEP-dependence of integrated tunnelling current for model transients with a gaussian spectral shape as a function of their pulse duration (FWHM), revealing a pronounced decrease in *I*_CEO_ when transitioning from single-cycle to multi-cycle waveforms. The calculations assume a simplified nonlinear current-field dependence of ${I}_{{\rm{CEO}}}\, \sim {E}+{E}^{3}$ (red curve) and ${I}_{{\rm{CEO}}}\, \sim {E}+{E}^{5}$ (pink). Source data

From the spectra of Fig. 1d, we reconstruct the temporal shape of the field of both pulses by an inverse Fourier transform up to a constant CEP offset. For τ = 0 fs, the superposition of both pulses synthesizes a waveform (Fig. 1e) with a full-width at half-maximum (FWHM) of the intensity envelope of 5.2 fs, corresponding to a single optical cycle centred at 190 THz. For ${\varphi }_{{\rm{CE}}}=0$, the resulting waveform exhibits a 1.36 ratio between the positive and negative field maxima. Numerical simulations confirm that the subcycle character of the near-field waveform in the tip–sample junction is preserved from the incident transient (Extended Data Fig. 3). In the diffraction-limited focus at the STM tip, peak electric fields of up to 7.6 MV cm^−1^ are reached (Methods). The inset in Fig. 1e illustrates two exemplary waveforms with ${\varphi }_{{\rm{CE}}}=0$ and ${\varphi }_{{\rm{CE}}}=\pi$. The acousto-optic phase shifter sweeps the CEP linearly in time as ${\varphi }_{{\rm{CE}}}\left(t\right)\propto 2\pi {f}_{{\rm{CEO}}}t$, where *f*_CEO_ ≈ 1 kHz is the carrier-envelope offset frequency (Methods). The CEP-dependent fraction of the tunnelling current, called ${I}_{{\rm{CEO}}}$, which is modulated at ${f}_{{\rm{CEO}}}$, is detected by lock-in demodulation, yielding also its phase ${\it{\phi }}_{{\rm{CEO}}}$ in relation to ${\varphi }_{{\rm{CE}}}\left(t\right)$.

## Fingerprints of subcycle tunnelling

**Fig. 2:**
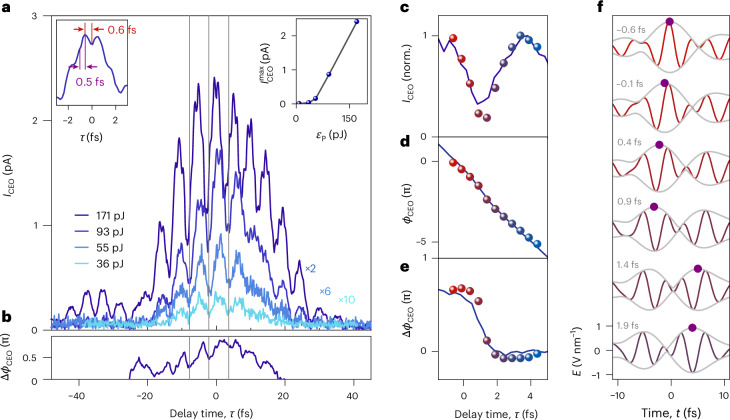
Evolution of lightwave-driven tunnelling currents with delay time ${\boldsymbol{\tau}}$. **a**, CEP-modulated tunnelling current ${I}_{{\rm{CEO}}}$ as a function of τ for various pulse energies *ε*_p_ = 171 pJ, 93 pJ, 55 pJ and 36 pJ (colour coded from dark to light blue, *I*_DC_ = 100 pA, *V*_B_ = 200 mV). For τ = 0 fs, the maxima of the envelopes of both NIR pulses coincide in time. Grey lines: positions of current minima for *ε*_p_ = 93 pJ. Datasets for low pulse energies are multiplied by a constant factor as indicated. Left inset: zoom-in of ${I}_{{\rm{CEO}}}$ for *ε*_p_ = 171 pJ. Red and purple vertical lines and arrows mark exemplary sub-femtosecond features. Right inset: nonlinear scaling of the peak value of ${I}_{{\rm{CEO}}}$(τ) with the pulse energy *ε*_p_. **b**, Phase difference $\Delta {\it{\phi} }_{{\rm{CEO}}}$ after subtracting a linear slope from the corresponding experimentally measured phase ${\it{\phi} }_{{\rm{CEO}}}$ (*ε*_p_ = 171 pJ). $\Delta {\it{\phi} }_{{\rm{CEO}}}$ exhibits steps at the positions of the current minima in **a** (grey vertical lines). **c**, Comparison of measured (solid line) and simulated (dots) CEP-modulated part of the tunnelling current as a function of τ. The experiments were taken above a Cu(111) surface with *ε*_p_ = 75 pJ and *V*_B_ = 0 mV (setpoint, *I*_set_ = 100 pA at *V*_B_ = 200 mV). **d**, Corresponding phase ${\it{\phi} }_{{\rm{CEO}}}$ of the experimental (solid line) and simulated (dots) tunnelling current. The theoretical data were shifted in τ, and a constant offset was applied to match the experimental data. **e**, Experimental (solid line) and simulated (dots) differential phase ${\Delta \it{\phi} }_{{\rm{CEO}}}$ obtained by subtracting a linear slope (−π/fs) from **d**. **f**, Electric field transients (coloured curves, colour code according to **c**–**e**) together with their respective envelopes (grey curves) for different τ. For certain τ, the maximum of the envelope (dots) exhibits sudden changes in time, explaining the phase jumps of ${\it{\phi} }_{{\rm{CEO}}}$ (see text). Source data

**Extended Data Fig. 4:**
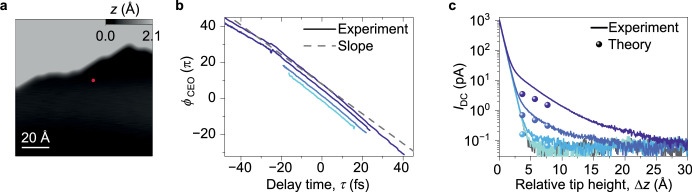
STM imaging, measured phase of the lightwave-driven current, and distance dependence of the DC tunnel current. **a**, Constant-current STM image of Ag(100). The tip position during the lightwave-current measurements (Fig. 2a) is indicated with a red dot. After each measurement, the sample was re-inspected to exclude topographic changes, confirming nondestructive measurement conditions. **b**, Measured $\it{\phi }_{{\rm{CEO}}}(\tau )$ of *I*_CEO_ for pulse energies of 171 pJ, 93 pJ, 55 pJ and 36 pJ (colour coded from dark to light blue). Phases for all pulse energies evolve in parallel. A vertical offset is applied for visual clarity. The slope (dashed grey line) follows ${\it{\phi }}_{{\rm{CEO}}}(\tau )$ for 171 pJ in a short interval between the two central minima in *I*_CEO_. **c**, DC component of the current measured simultaneously to ${I}_{{\rm{CEO}}}$ in Fig. 4a. Measurement taken at τ  = 0, with a bias voltage of *V*_B_ = 200 mV and a current setpoint ${I}_{{\rm{DC}}}$ = 1 nA, for the pulse energies 171 pJ, 93 pJ, 55 pJ and 36 pJ (colour coded from dark to light blue). For all measurements, ${I}_{{\rm{DC}}}(\Delta z)$ features a short-range decay with a decay constant of one order of magnitude over 1.07 Å. This is characteristic of steady-state STM and thus also observed in the absence of lightwaves (grey curve, largely hidden by overlap). For large pulse energies *ε*_p_ > 55 pJ, ${I}_{{\rm{DC}}}$ shows an additional long-range decay component with decay constants *κ* = 0.15 Å^−1^ and 0.2 Å^−1^ for 171 pJ and 93 pJ, respectively. This supports the theoretical finding of tunnelling of excited electrons through a reduced barrier. The blue dots represent the simulated rectified current ${I}_{{\rm{DC}}}(\tau )$ for a delay time of τ = 3.4 fs for absolute tip-sample distances of *d*_st_ = 14 Å, 16 Å and 18 Å and field strengths of 1.04 V/nm, 0.76 V/nm and 0.59 V/nm (dark to light blue). The dots are shifted in *z* by 10.3 Å and multiplied by a constant factor of 220 to display them on the same axis as the experimental data. In the simulations, *z* is the distance between the centre of the foremost tip atom and the topmost atomic plane of the surface cluster (defined by the centre of atoms). Consequently, the actual tunnelling gap is smaller by twice the atomic radius in the calculations. By contrast, in the experiment Δ*z* refers to the change in tip height with respect to the set-point parameters (1 nA, 200 mV), such that Δ*z* = 0 indicates a finite tunnelling gap of several Å. Therefore, the offset of 10.3 Å between *d*_ts_ and Δ*z* accounts for these two differences. Source data

With this novel scheme, which avoids artefactual signals, we search for genuine lightwave-driven tunnelling currents on an atomically flat Ag(100) surface (Extended Data Fig. 4). Importantly, a stable and highly reproducible CEP-dependent component of the tunnelling current emerges (Fig. 2a), proving the existence of subcycle charge transfer. The stroboscopically measured currents ${I}_{{\rm{CEO}}}\left(\tau \right)$ for four pulse energies *ε*_p_ = 171 pJ, 93 pJ, 55 pJ and 36 pJ are qualitatively similar but scaled in amplitude. ${I}_{{\rm{CEO}}}\left(\tau \right)$ occurs only in the vicinity of τ  = 0 fs, where the two pulses overlap to form the most asymmetric waveforms (Extended Data Fig. 2). Most remarkably, ${I}_{\mathrm{CEO}}\left(\tau \right)$ exhibits strong oscillations on a subcycle scale, and reproducible structures down to sub-femtosecond scales (Fig. 2a, left inset), which proves the presence of attosecond current components^33,36,37,39,43,44^. The threshold-like scaling of the maxima of *l*_CEO_(τ) with *ε*_p_ (Fig. 2a, right inset) underpins the nonlinear character of the tunnelling process.

Notably, the subcycle dynamics also manifest in the phase of the tunnelling current. For better visibility, Fig. 2b shows ${\Delta \it{\phi} }_{{\rm{CEO}}}(\tau )$ obtained by subtracting a linear slope from ${\it{\phi} }_{{\rm{CEO}}}(\tau )$ (Extended Data Fig. 4). ${\Delta \it{\phi} }_{{\rm{CEO}}}(\tau )$ exhibits steps at the positions of the minima in ${I}_{{\rm{CEO}}}\left(\tau \right)$ (vertical lines). As we will show below, this structure relates to the NIR carrier waveforms and their change with τ. The CEP and waveform dependence of the current clearly verifies attosecond tunnelling^33,36,37,39,43,44^. On this timescale, electrons are expected to exhibit a non-instantaneous response that could also non-trivially convolve temporal and spatial signatures of the wavefunction.

## Full quantum theory of NIR-induced tunnelling

While the presence of subcycle, attosecond current components is directly evident from the experimental observables, we compare our results with a full real-space/time-domain quantum theory to microscopically model these processes. We simulated the light-induced charge transfer between two atomic sodium clusters representing the tunnelling junction with time-dependent density functional theory (TD-DFT; Methods), which treats strong-field and multi-photon physics on equal footing^45,46^. The theoretically retrieved CEP-dependent modulation depth ${I}_{{\rm{CEO}}}(\tau)$ and its phase ${\it{\phi} }_{{\rm{CEO}}}(\tau )$ faithfully reproduce the experimental data (Fig. 2c–e). On the basis of the excellent theory–experiment agreement, we use the TD-DFT results to extract the exact temporal shape of the tunnelling current.

The strong dependence of ${I}_{{\rm{CEO}}}$ on τ (Fig. 2c) is caused by the sensitivity of the electronic wave packet dynamics to the precise shape of the NIR carrier field. Figure 2f shows the τ-dependent optical waveforms that drive the current. The peak of ${I}_{{\rm{CEO}}}$ (τ  = −0.6 fs) is caused by the single-cycle transient in Fig. 2f, where the influence of the CEP on light-driven currents is maximal (Extended Data Fig. 3). For τ  = −0.1 fs, the carrier-wave maximum is shifted against the envelope maximum, creating a CEP offset, which gradually grows with τ and underlies the linear shift in ${\phi }_{\mathrm{CEO}}(\tau)$. For τ  ≥ 0.4 fs, an oscillation node shifts through the pulse eventually destroying the single-cycle character. At τ  = 0.9 fs and 1.4 fs, the envelope features two almost equally strong envelope maxima (purple dots). Under this condition, the CEP has a minimal effect on the tunnelling current, explaining the dip in ${I}_{{\rm{CEO}}}$. For 0.9 fs <  τ < 1.4 fs, the maximum shifts from before to after the node. This sudden CEP change manifests as a step in the experimental and computed ${\it{\phi} }_{\mathrm{CEO}}(\tau )$, occurring within a few hundred attoseconds in τ (Fig. 2e).

## Time and space uncertainty

**Fig. 3:**
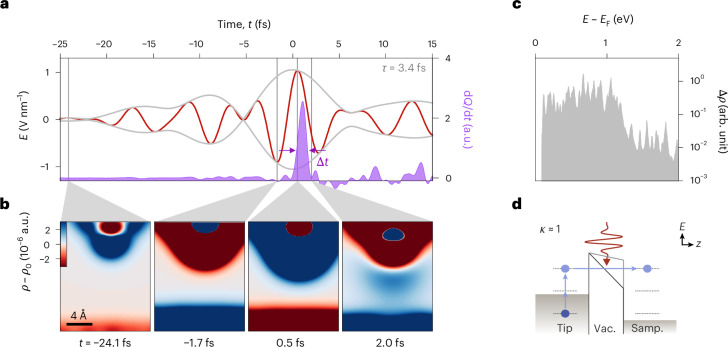
Time-domain DFT simulation of subcycle currents. **a**, Electric field transient (red) and its envelope (grey) of an exemplary single-cycle transient (τ  = 3.4 fs, ${\varphi }_{{\rm{CE}}}=0$ and peak field, 1.04 V nm^−1^) used for the TD-DFT simulations of lightwave-driven currents between two atomic sodium clusters. The corresponding simulated current transient $I(t)={\rm{d}}Q/{\rm{d}}t$ (purple) is confined to a duration of Δ*t* = 988 as (FWHM). **b**, Selected snapshots of the spatial distribution of the simulated relative charge density $\Delta \rho \left(t\right)=\rho \left(t\right)-{\rho }_{0}$ (colour coded in atomic units) in the tunnel junction (tip–sample distance 16 Å), with the equilibrium charge density ${\rho }_{0}$. The images represent a cut through the middle of the tip and the sample at *y* = 0. Emerging excitations at *t* = −24.1 fs follow the geometry of the sodium clusters forming tip (top) and sample (bottom). At *t* = −1.7 fs and 0.5 fs, a strong shake-up of $\Delta \rho \left(t\right)$ becomes apparent, while $\Delta \rho$ inside the tunnelling gap indicates the rapid charge transfer at *t* = 2.0 fs. The colour scale saturates at 3 × 10^−6^ atomic units (a.u.). **c**, Simulated energy-resolved change in occupation of electronic states after an excitation of the tip–sample cluster with a Gaussian pulse (peak electric field, 2.16 V nm^−1^; centre frequency, 242 THz). States above the Fermi energy ${E}_{{\rm{F}}}$ get transiently occupied. **d**, In the photon-assisted lightwave-driven tunnelling regime (Keldysh parameter $\kappa \approx 1$), electrons (blue dots) tunnel through the vacuum barrier (Vac.) from the tip to the sample (Samp.) via pathways (blue arrows) including excited states (dashed lines) but still see the light-field modulation (red) of the barrier. Source data

With the full quantum theory at hand, we can further follow the wave packet in space and time while an exemplary field transient (Fig. 3a) biases the junction. Figure 3b shows four snapshots of the simulated tip–sample junction during the charge transfer process. The difference in charge density $\Delta \rho (t,x,z)$ with respect to the unperturbed ground state ${\rho }_{0}$ is colour coded (see Supplementary Information for full movie). When the electric field is still low (*t* = −24.1 fs), $\Delta \rho$ reflects the geometry of tip and sample. During the main negative (*t* = −1.7 fs) and positive (*t* = 0.5 fs) half cycles, a strong shake-up of $\Delta \rho \left(t\right)$ evolves, with Δ*ρ* occupying increasingly more volume. At *t* = 2.0 fs, the wave packet bridges the gap as indicated by the finite $\Delta \rho$ between tip and sample.

The computed time derivative of the corresponding charge transfer represents the tunnelling current (Fig. 3a, purple), which flows within a time window of slightly less than 1 fs. Most remarkably, the current maximum in Fig. 3a is distinctly delayed by 0.5 fs after the field maximum and persists up to about 1.5 fs. Unlike in THz-STM on the 10–100 fs scale, the current response in attosecond STM is no longer instantaneous as the speed of the barrier modulation starts to reach the response time of the electrons. The retardation of the charge transfer to the field, which is exclusively revealed by our quantum simulations, is characteristic of the intermediate light–matter interaction regime, *κ* ≈ 1, and related to the Keldysh time^32^. Moreover, the increasing evanescent $\Delta \rho$ extending into the vacuum region (Fig. 3b) hints towards a transient occupation of excited states that lie energetically close to the vacuum level. This is confirmed by the simulated change in occupation (Fig. 3c). Hence, tunnelling at *κ* ≈ 1 occurs as sketched in Fig. 3d: when the electric field of the single-cycle pulse (red) tilts the potential between tip and sample, electrons cross the barrier non-adiabatically via quantum pathways that include excited states in tip and sample. These spatially more extended states, which face reduced tunnelling barriers, can be reached by one- or few-photon excitations.

Remarkably, the tunnelling barrier is extremely thin, irrespective of the light field. This scenario differs profoundly from the usual situation encountered in strong-field-driven isolated atoms or tunnelling–photoemission from a single electrode, where the width of the triangular tunnelling barrier scales inversely with the bias field, and the relative strengths of multi-photon and tunnelling processes are gauged by the Keldysh parameter *κ* (ref. ^32^). In our experiment, the tunnelling process can occur in the strong-field regime or by directional currents shaped through the interference of few-photon excitation processes of different orders^47^. In any case, such photon-assisted tunnelling is susceptible to the phase of the junction modulation, that is, the CEP and waveform of the electric field transient. Photon-assisted lightwave-driven tunnelling introduces a finite temporal delay, a spatial spread of the wavefunction and a strongly enhanced tunnelling probability. Therefore, this scenario closely intertwines temporal signatures with the spatial structure.

## Measuring the size of the attosecond wave packet

**Fig. 4:**
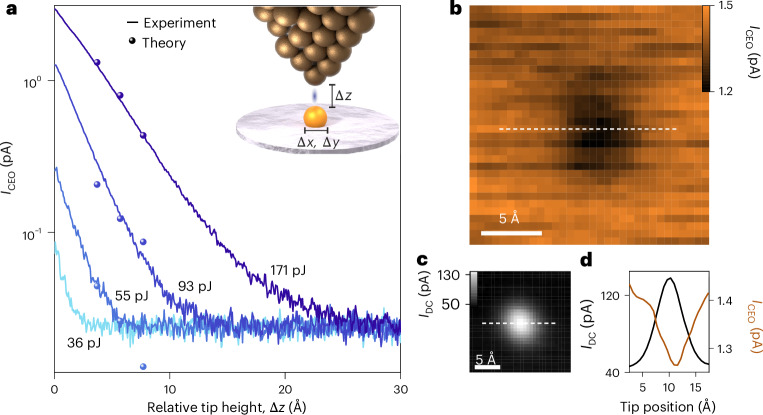
Atomically resolved subcycle currents. **a**, Measured ${I}_{{\rm{CEO}}}$ (solid lines) as a function of tip–sample distance Δz for four representative pulse energies of 171 pJ, 93 pJ, 55 pJ and 36 pJ (colour coded from dark to light blue as in Fig. 2a). Simulated ${I}_{{\rm{CEO}}}(\Delta z)$ (dots) for peak fields of 1.04 V nm^−1^, 0.76 V nm^−1^ and 0.59 V nm^−1^ (colours as in corresponding experimental data). For *ε*_p_ = 171 pJ, the decay length of ~8.7 Å reflects tunnelling through a shallow remaining barrier. For *ε*_p_ < 100 pJ, the decay becomes much steeper (<5 Å). The background is given by the measurement noise floor. Inset: experimental geometry of the tunnel junction. **b**, Image of a single Cu adatom on Ag(100) recorded with attosecond tunnelling currents proving atomic confinement (*ε*_p_ = 93 pJ). **c**, Simultaneously recorded image obtained from ${I}_{{\rm{DC}}}$. **d**, Line cut through **b** and **c** (dashed lines) confirming a similar lateral resolution for DC and lightwave-induced currents. Source data

Our combination of sub-atomic real-space resolution and attosecond temporal definition with the well-defined environment of ultrahigh vacuum allows us to experimentally assess the space-time limit of single-electron tunnelling. We first measured the vertical decay of the electron wave packet in the junction, that is, the distance dependence of ${I}_{{\rm{CEO}}}$ (Fig. 4a) by setting τ to the maximum of ${I}_{{\rm{CEO}}}$ and retracting the tip from the sample starting from a defined setpoint (*I*_DC_ = 1 nA at *V*_B_ = 200 mV). For the highest pulse energy of 171 pJ, ${I}_{{\rm{CEO}}}$ decays by one order of magnitude within *l*_c_ = 8.7 Å. This decay constant is distinctly larger than characteristic of steady-state tunnelling at the Fermi level (*l*_c_ = 1 Å; Extended Data Fig. 4) and attests to the contribution of excited electrons with an effectively reduced barrier and more extended wavefunctions.

**Extended Data Fig. 5:**
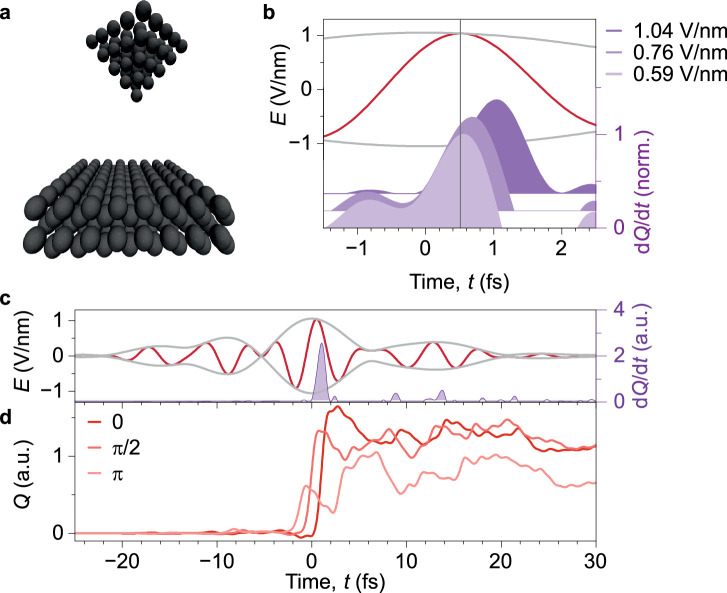
Model system and TD-DFT simulations of lightwave-driven charge transfer. **a**, Tip–sample junction model composed of sodium atoms. The tip is represented by a tetragonal pyramid consisting of 55 Na atoms, while the sample is modelled by a Na(100) slab with 256 atoms arranged in four atomic layers. **b**, Time-domain DFT simulations of subcycle currents for different peak electric field strengths. The electric field transient (red) and its envelope (grey) of an exemplary single-cycle pulse (τ  = 3.4 fs, ${\varphi }_{{\rm{CE}}}=0$, peak field 1.04 V/nm) are shown; the field maximum is indicated by the vertical grey line. The normalized instantaneous current ${\rm{d}}Q/{\rm{d}}t$ is shown for three field strengths (1.04, 0.76, and 0.59 V/nm; dark to light purple). Curves are vertically offset for clarity. **c**, Simulated carrier-envelope-phase (CEP)–dependent charge transfer between tip and sample for a field strength of 1.04 V/nm (larger time window compared to **b**). The current transient is confined to a sub-femtosecond duration (Δ*t* = 988 as FWHM for ${\varphi }_{{\rm{CE}}}=0$). **d**, The corresponding time-resolved charge transfer *Q(t)* is shown for ${\varphi }_{{\rm{CE}}}=0$, π/2 and π, revealing a pronounced CEP dependence of the charge transfer step near *t* = 0 fs. Source data

The simulated ${I}_{{\rm{CEO}}}(\Delta z)$ faithfully reproduces the experimental decay length. Remarkably, ${l}_{{\rm{c}}}$ can be substantially reduced to 5.8 Å, 4.3 Å and 3.8 Å, by decreasing the NIR pulse energy to 93 pJ, 55 pJ and 36 pJ, respectively. This field-dependent variation of the decay length is similar to the amplitude of the Kramers–Henneberger shift^2^ obtained for our experimental parameters (Methods). Our quantum theory (Fig. 4a, dots) confirms these differences to originate from a reduction of few-photon excitation, which would otherwise delay and delocalize electrons (Extended Data Fig. 5). Thus, by optimizing control parameters, the spatio-temporal sweet spot of sub-femtosecond tunnelling confined to Ångstrom scales in vertical direction is reached.

To experimentally access the critical lateral spread of the wavefunction, we map ${I}_{{\rm{CEO}}}$ above a single copper adatom on Ag(100). At a pulse energy of *ε*_p_ = 93 pJ, the tip was approached above the centre of the atom to *I*_set_ = 100 pA at *V*_B_ = 200 mV and subsequently raster-scanned in constant-height mode, which excludes artefacts from a potential crosstalk between the DC and lightwave-driven currents via the STM feedback loop (Methods). The simultaneously recorded maps of ${I}_{{\rm{CEO}}}$ and ${I}_{{\rm{DC}}}$ (Fig. 4b,c) clearly resolve the atom; line profiles confirm the atomic lateral confinement within ~6 Å (Fig. 4d), proving that the sub-femtosecond electron tunnelling transients are spatially localized on the atomic scale. Notably, ${I}_{{\rm{CEO}}}$ decreases above the adatom. The exact contrast mechanism is currently under investigation. We tentatively assign it to the local decrease in the work function of the sample above the adatom^48^, potentially leading to a locally more symmetric tunnelling barrier that reduces ${I}_{{\rm{CEO}}}$. Independently of the detailed atomistic contrast mechanism, tunnelling is merely determined by the wavefunction overlap between the initial and final states. Therefore, our atomically resolved images demonstrate that attosecond electron pulses can be sculpted into few-Å wave packets in all three spatial dimensions.

## Conclusion

Our attosecond/atomic-scale lightwave STM allows us to resolve the intrinsic space-time limit of electronic wave packets. In a novel CEP-controlled two-colour pump–probe-like experiment, we study how temporal driving of an electronic wavefunction across a tunnelling junction affects its spatial shape. CEP- and delay time-dependent tunnelling currents reveal attosecond electron motion, whose microscopic dynamics are set by photon-assisted tunnelling. The simultaneous action of few-photon and strong-field aspects leads to a fascinating correlation between the delayed timing of the tunnelling electrons and their spatial extent. In the future, absolute clocking experiments may further clarify the role of attosecond delays in field-driven tunnelling. By tuning the field strength, the electron wave packets can be confined to below 1 fs in time and few Å in all three spatial dimensions. This breakthrough not only enables us to probe the wavefunction’s extension by imaging a single Cu adatom with attosecond currents; it also transforms our ability to study and control quantum phenomena in molecules, nanoscale devices and correlated materials. Exciting foundational dynamics that may now become accessible range from quantum electrodynamic corrections to the Kramers–Henneberger transformation^7^, signatures of chaos^49^ and dynamical transparencies of tunnelling barriers^50^ to spatio-temporal correlations of electronic wavefunctions during the making or breaking of chemical bonds^8^.

## Methods

### NIR pump–probe scheme

The femtosecond Er:fibre laser system is based on an offset-free frequency comb (TOPTICA DFC CORE), which outputs a CEP-stable pulse train at a repetition rate of 80 MHz and a central wavelength of 1,560 nm (refs. ^51,52^). The CEP control is achieved via an acousto-optic phase shifter (AOPS)^53^. The repetition rate of the laser ${f}_{{\rm{rep}}}$ and the radio frequency of the AOPS driver ${f}_{{\rm{AOPS}}}$ are locked to the same reference clock. Detuning ${f}_{{\rm{AOPS}}}$ from ${f}_{{\rm{rep}}}$ such that ${f}_{{\rm{AOPS}}}={f}_{{\rm{rep}}}$ + ${f}_{{\rm{CEO}}}$ creates a linear CEP slip $\Delta {\varphi }_{{\rm{CE}}}=2\uppi {f}_{{\rm{CEO}}}t$. Subsequently, a dual-stage Er:fibre amplifier boosts the power to 400 mW, 70% (280 mW) of which are used for continuum generation in a highly nonlinear optical fibre. Dispersion compensation is achieved separately for frequency components above and below 1,560 nm by means of a two-branch prism compressor. Razor blades in the Fourier plane of both beam paths block the more strongly structured spectral parts near the central frequency. The end mirror of the high-frequency prism compressor branch is mounted on a closed-loop piezoelectric stage with nanometre precision. This is used to adjust the relative arm length and therefore the pump–probe delay time τ between the two pulses. The temporal overlap is monitored with spectrally resolved sum frequency generation in a 10 µm thin type-I β-barium borate crystal. After compression, the pulse energy is tunable between 36 pJ and 171 pJ without altering the spectral content or changing the dispersion, using custom-built reflective neutral density filters consisting of thin layers of gold on fused silica. A mirror telescope including a pinhole in the focus serves to clean the spatial mode and expands the beam for improved focusing in the STM. A 90° periscope rotates the polarization vertically to align the polarization with the tip axis. We performed FROG characterizations of the dispersive and solitonic parts of the spectrum after the mode cleanup and obtain an overall uncertainty of 10% for the pulse duration (FWHM), leading to a pulse duration of 5.2 fs ± 0.5 fs, for the combined ultrashort pulse.

### Attosecond waveform control

The intensity map of Extended Data Fig. 2 shows the envelope of the field ${E}_{{\rm{env}}}$ as a function of time *t* and τ, illustrating how the superposition of the two pulses modulates ${E}_{{\rm{env}}}$. As τ varies, the field envelope ${E}_{{\rm{env}}}(t)$ alternates between single- and multi-cycle waveforms. Accordingly, the maximum of the field envelope ${E}_{{\rm{env}}}^{\max }$ oscillates with τ and the carrier phase at the pulse maximum ${\it{\phi} }_{\max }$ exhibits a dominantly linear dependence on τ (Extended Data Fig. 2).

### Power stability of the dual pulse set-up

The indispensable power stability of the synthesized dual pulse train while sweeping τ was monitored with a photodiode (Extended Data Fig. 2). While the CEP modulation scheme is active, the measured average power P(τ) remains constant (c, black curve). The demodulated component of the power ${P}_{{\rm{CEO}}}$ at ${f}_{{\rm{CEO}}}$ (grey curve) reveals a minute relative modulation (standard deviation 10.2 nW), which however does not depend on τ.

### STM set-up and sample preparation

All measurements were performed inside an ultrahigh-vacuum chamber (base pressure, *p* < 2 × 10^−10^ mbar) equipped with a custom-built low-temperature STM operated at a base temperature of ~5 K (lHe). The bias voltage is applied to the sample and the tip is grounded. The tunnelling current is collected from the tip, routed through a coaxial cable (length 1.2 m) and amplified with a gain factor of *G* = 1 × 10^9^ V A^−1^ at a bandwidth of 1.1 kHz (FEMTO Messtechnik GmbH, DLPCA-200, at room temperature). We use an electro-chemically etched tungsten tip. The laser enters the STM chamber through a diamond window. A custom-designed in situ parabolic mirror with a large numerical aperture of 0.6 and an effective focal length *f* = 12.5 mm focuses the collimated laser beam onto the STM tip. We measure a focal spot diameter of *w*_0_ = 4.3 µm, leading to a maximal field strength of 7.6 MV cm^−1^ of the single-cycle pulse (low frequency pulse, 4 MV cm^−1^; high frequency pulse, 3.7 MV cm^−1^).

The parabolic mirror can be positioned with stick-slip piezo motors to optimize the focal spot on the tip–sample junction. To minimize dispersion, no windows are placed in the thermal shields. Instead, a custom-designed series of apertures is used to minimize heating owing to black-body radiation entering through the diamond window. The NIR laser light reflected off the sample is re-collimated by the wide-angle parabolic mirror and directed out of the scan head to reduce thermal load. Coarse positioning and scanning were achieved by moving the sample relative to the tip, thereby maintaining the position of the laser focus at the tip apex during STM operation. The Ag(100) sample is cleaned by sputter and annealing cycles. Cu adatoms were deposited in situ onto the sample inside the scan head at a temperature of ~5 K.

### Sensitivity of STM to laser power modulations

We quantify the effect of laser power modulations on the junction stability by focusing the train of femtosecond laser pulses (repetition rate, 80 MHz; centre wavelength, 1,560 nm) onto the junction as in the primary experiments. Slight variations of the pulse energy (mean value, 106 pJ) with an acousto-optic modulator (frequency, 874 Hz; see Extended Data Fig. 1) introduce prominent modifications of the tunnelling current, which can be identified as thermally induced modulations ${I}_{{\rm{th}}}$ of the total direct current ${I}_{{\rm{DC}}}$. ${I}_{{\rm{th}}}$ scales linearly with the power modulation depth (STM feedback loop with *I*_DC_ = 100 pA at *V*_B_ = 200 mV), indicating that the thermal current modulation ${I}_{{\rm{th}}}/{I}_{{\rm{DC}}}$ even exceeds the relative power modulation $\Delta P/{P}_{0}$. Most critically, for finite ${V}_{{\rm{B}}}$, ${I}_{{\rm{DC}}}$ is much larger than the coveted lightwave-driven current. Because ${I}_{{\rm{th}}}$ is synchronized with power modulations, it is easily confused with lightwave-driven currents. This highlights the need of extraordinary stability of the relative power better than 10^−4^.

### Measurement of CEP-modulated tunnelling currents as a function of delay time

${\varphi }_{{\rm{CE}}}$ was modulated at *f*_CEO_ = 917 Hz, which is within the bandwidth of the current pre-amplifier. This frequency was optimized to minimize detection noise. A lock-in amplifier demodulates the total tunnelling current at ${f}_{{\rm{CEO}}}$ extracting the resulting CEP-dependent fraction ${I}_{{\rm{CEO}}}$. For the data shown in Fig. 2a, ${I}_{\mathrm{CEO}}\left(\tau \right)$ was recorded for 1,001 delay steps over a range of 160 fs with an integration time of 100 ms (*ε*_p_ = 55 pJ and 36 pJ, 501 steps over 80 fs, integration time 300 ms). To keep the tip–sample separation constant during the delay scan, the measurements were performed in constant current mode with a DC tunnelling current setpoint of 100 pA and at a bias voltage of 200 mV. The lock-in signal was divided by the gain factor *G* (10^9^ V A^−1^) to convert it to a current.

### Distance dependence measurement

To measure ${I}_{{\rm{DC}}}(\Delta z)$ and ${I}_{{\rm{CEO}}}(\Delta z)$ as a function of relative tip–sample distance Δz, we set *f*_CEO_ = 917 Hz and set a lock-in integration time of 10 ms. Δz was swept from 0 Å to 30 Å over 35 s at *V*_B_ = 200 mV. The reference position Δ*z* = 0 was set at *I*_DC_ = 1 nA at *V*_B_ = 200 mV before each measurement. For every pulse energy, 30 consecutive distance sweeps were averaged to improve the signal-to-noise ratio. The delay τ was set to the respective maximum of ${I}_{\mathrm{CEO}}(\tau )$ observed in Fig. 2a.

### Attosecond-STM image of a single Cu adatom

We scanned the sample in constant-height mode, after switching off the feedback loop above the centre of the Cu adatom at a setpoint of *I*_DC_ = 100 pA at *V*_B_ = 200 mV. For each pixel, we recorded ${I}_{{\rm{DC}}}(x,y)$ and ${I}_{{\rm{CEO}}}(x,y)$ simultaneously. We used a lock-in integration time of 100 ms and a scanning speed of 311 ms per pixel (image size 32×32 pixels). The pulse energy was set to *ε*_p_ = 93 pJ, and we modulated the CEP at ${f}_{{\rm{CEO}}}=$ 917 Hz.

### Simulation of quantum mechanical wave packet ${\boldsymbol{\psi }}({\boldsymbol{x}},{\boldsymbol{t}})$

For Fig. 1a,b, we simulated the scattering process of a quantum mechanical Gaussian wave packet $\psi \left(x,t\right)$ with a rectangular potential barrier *V*(*x*) that is modulated with a spatially homogeneous, time-dependent electric field ${E}_{x}\left(t\right)$. The influence of the tilt of the potential landscape induced by ${E}_{x}\left(t\right)$ is fully described by a purely horizontal time-dependent shift of the still rectangular barrier by the so-called Kramers–Henneberger transformation^2^. Using this unitary gauge transformation, we obtain the following time-dependent, one-dimensional Schrödinger equation:

$$i\hslash \frac{\partial \psi \left(x,t\right)}{\partial t}=-\frac{{\hslash }^{2}}{2{m}_{e}}\frac{{\partial }^{2}\psi \left(x,t\right)}{{\partial x}^{2}}+V\left(x-\frac{e{E}_{x}\left(t\right)}{{m}_{e}{\omega }^{2}}\right)\psi \left(x,t\right),$$

with the electron mass ${m}_{e}$, the elementary charge e and the central angular frequency ω. In this gauge, the position of the potential barrier shifts in time proportional to the electric field by $\Delta x(t)=\,\frac{e{E}_{x}\left(t\right)}{{m}_{e}{\omega }^{2}}$. The above Schrödinger equation is solved numerically using the Crank–Nicolson scheme^54^. For the presentation of the results in Fig. 1a,b, the coordinate system is transformed back to Cartesian coordinates. For the experimental parameters of $\omega =2\uppi \times 200\,{\rm{THz}}$, ${E}_{x}^{\max }$ = 1 V nm^−1^, the maximal excursion Δx of the barrier amounts to $1.1\,{{\AA }}$.

### Time-dependent DFT simulations

A tip–sample junction is modelled using a 55-Na-atom tetragonal pyramid for the tip and a (100) surface consisting of 256 Na atoms (4 atomic layers), as shown in Extended Data Fig. 5. The use of Na reduces the computational cost, and similar simulations have previously been shown to successfully reproduce key aspects of the response of transition-metal systems such as copper and tungsten at comparable energy scales^55^. The distance between the atomic clusters is defined as the distance between the apex atom of the tip and the topmost layer of the slab.

The electronic structure of the system is described using DFT with the LDA functional and the Troullier–Martins pseudopotential, in a grid basis set with a grid spacing of 0.2 Å, using the Octopus code^56^. For the real-time propagation of the Kohn–Sham wavefunction, we have used an enforced time-reversal propagator, with a time step $\Delta t=\,$0.075 atomic units (1 atomic unit = 24.2 as). The system is driven by two laser pulses polarized along the *z*-axis. We refer to them as the dispersive wave (dw) and soliton (st) and express them in terms of a superposition of Gaussian waveforms:

$${E}_{\mathrm{dw}}\left(t\right)=\mathop{\sum }\limits_{{\rm{i}}=1}^{3}{a}_{{\rm{i}},\mathrm{dw}}{{\rm{e}}}^{-{\left(\frac{t-{t}_{0}-{b}_{{\rm{i}},\mathrm{dw}}+\frac{\Delta \tau }{2}}{{c}_{{\rm{i}},\mathrm{dw}}}\right)}^{2}}\cos \left({d}_{{\rm{i}},\rm{{dw}}}\left(t-{t}_{0}+\frac{\Delta \tau }{2}\right)+{\phi }_{{\rm{i}},\rm{{dw}}}+\Delta {\varphi }_{\rm{{CE}}}\right)$$

$${E}_{\mathrm{st}}\left(t\right)=\mathop{\sum }\limits_{{\rm{i}}=1}^{3}{a}_{{\rm{i}},\mathrm{st}}{{\rm{e}}}^{-{\left(\frac{t-{t}_{0}-{b}_{{\rm{i}},\mathrm{st}}-\frac{\Delta \tau }{2}}{{c}_{{\rm{i}},\mathrm{st}}}\right)}^{2}}\cos \left({d}_{{\rm{i}},\rm{st}}\left(t-{t}_{0}-\frac{\Delta \tau }{2}\right)+{\phi }_{{\rm{i}},\rm{st}}+\Delta {\varphi }_{\rm{CE}}\right).$$

The parameters *a*_i_, *b*_i_, *c*_i_, *d*_i_ and $\it{\phi }_{{\rm{i}}}$ were optimized to fit the shape of the retrieved experimental pulses obtained by the inverse Fourier transform of the complex spectra (Fig. 1d).

The simulation time window *t* starts at t= 0 fs and lasts for 100 fs. τ and $\Delta {\varphi }_{{\rm{CE}}}$ are the delay time between the pulses and the CEP, respectively. For τ= 0, both pulses are centred at ${t}_{0}$. In the simulation, τ is implemented by shifting both pulses in opposite directions by $\pm \tau$/2, while in the experiment, only the dispersive wave is delayed by τ. We further included an extra envelope function to the total electric field:

$${E}_{z}\left(t\right)=\left({E}_{\mathrm{dw}}\left(t\right)+{E}_{\mathrm{st}}\left(t\right)\right)\times {e}^{-{\left(\frac{t-{t}_{0}}{\sigma }\right)}^{2}},$$

where σ=20 fs. This function ensures a smooth onset of the laser pulse, leading to stable dynamics while preserving the main features of the pulse.

The transferred charge Q(t) is defined as

$$Q\left(t\right)=\mathop{\iiint }\limits_{{V}_{\mathrm{slab}}}\rho \left({\mathbf{r}},t\right)-\rho \left({\boldsymbol{r}},0\right)d{\mathbf{r}},$$

where $\rho ({\mathbf{r}},t)$ corresponds to the diagonal terms of the density matrix projected on the grid basis, as a function of time. The slab volume ${V}_{{\rm{slab}}}$ is defined as the region between the bottom plane of the simulation box and a plane halfway between the two structures.

### Simulating CEP-dependent currents

**Extended Data Fig. 6:**
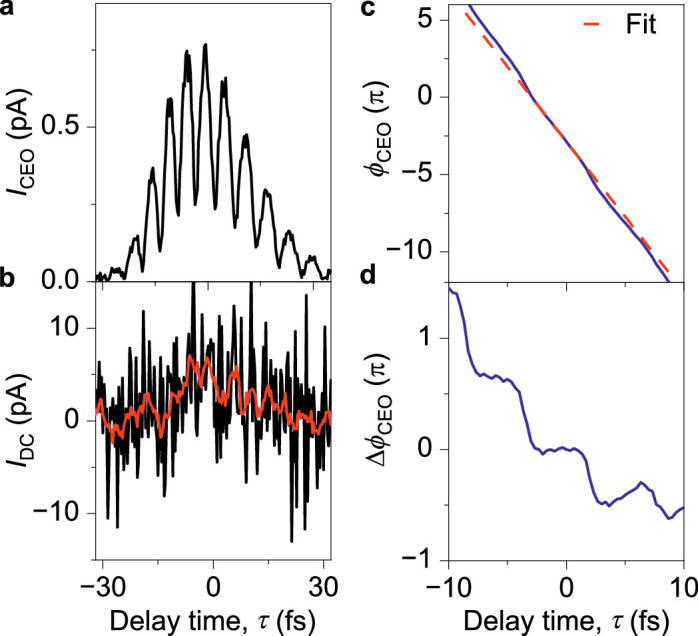
Comparison of *I*_CEO_ and *I*_DC_ measured phase *ϕ*_CEO_ and phase difference Δ*ϕ*_CEO_. **a**, CEP–modulated tunnelling current ${I}_{{\rm{CEO}}}$ as a function of τ measured above a Cu(111) surface for a pulse energy of *ε*_p_ = 75 pJ (*V*_B_ = 0 mV, setpoint at *I*_DC_ = 100 pA and *V*_B_ = 200 mV). **b**, Corresponding DC tunnelling current. The small increase in *I*_DC_ at τ = 0 fs points towards a rectified component of the lightwave-driven current. The red line shows the mean value over 10 consecutive datapoints. **c**, The measured phase ${{\it{\phi }}}_{{\rm{CEO}}}$ corresponding to ${I}_{{\rm{CEO}}}$ in **a** is shown, with a linear fit between two steps (red dashed line). **d**, The phase difference $\Delta {{\it{\phi }}}_{{\rm{CEO}}}$ is obtained by subtracting the linear fit from ${{\it{\phi }}}_{{\rm{CEO}}}$. The resulting $\Delta {{\it{\phi }}}_{{\rm{CEO}}}$ reveals distinct steps at the delay values of the minima of *I*_CEO_. Source data

For a given τ and tip–sample distance, we calculate $Q(t,\,{\varphi }_{{\rm{CE}}})$ for three different CEP values (${\varphi }_{{\rm{CE}}}=$ 0, π/2, π; see Extended Data Fig. 5). At late times, $Q\left(t\to \infty \right)$ becomes stationary and resembles the amount of transferred charge per pulse. We determine $Q\left(t\to \infty \right)$ by averaging over the last 200 data points of Q(t). Finally, we extract the CEP-dependent charge transfer $\Delta {Q}_{\mathrm{CEP}}\left(\tau \right)$ and the phase ${\phi }_{\mathrm{CEO}}(\tau )$ by fitting

$$f\left({\varphi }_{{\rm{CE}}}\right)={\Delta Q}_{{\rm{CEP}}}\cos \left({\varphi }_{{\rm{CE}}}+{\phi }_{{\rm{CEO}}}\right)+{Q}_{0}$$

to $Q\left(t\to \infty ,\,{\varphi }_{{\rm{CE}}}\right)$ with a constant offset ${Q}_{0}$. $\Delta {Q}_{{\rm{CEP}}}$ multiplied with the repetition rate corresponds to the current ${I}_{{\rm{CEO}}}(\tau )$. We repeat this procedure for a series of 11 delay times τ from −2.5 fs to 2.5 fs with a spacing of 0.5 fs. Owing to the Nyquist criterion, it is sufficient to simulate three values of ${\varphi }_{\mathrm{CE}}$. The result is plotted in Fig. 2c–e. Extended Data Fig. 6 shows the full measurement data of Fig. 2c–e. The theoretical data was shifted for clarity along the delay axis by 1.9 fs.

### Simulating the distance dependence of ${{\boldsymbol{I}}}_{{\bf{CEO}}}$

To calculate the distance dependence of ${I}_{{\rm{CEO}}}$ shown in Fig. 4a, we performed the above-described steps for a given field strength for three different centre-to-centre distances *z* between the foremost atoms forming the tunnelling junction (14 Å, 16 Å and 18 Å). The largest field strength of 1.04 V nm^−1^ corresponds to the estimated field strength at a pulse energy of 171 pJ for a 4-µm-wide focal spot. The relative scaling of the field strength used in the simulation 1.04/0.76 = 1.37 and 1.04/0.59 = 1.76 corresponds to the relative scaling of the pulse energies $\frac{\sqrt{171\,{\rm{pJ}}}}{\sqrt{93\,{\rm{pJ}}}}=1.36$ and $\frac{\sqrt{171\,{\rm{pJ}}}}{\sqrt{55\,{\rm{pJ}}}}=1.76$. Simulated data points are all shifted by a constant *z*-offset of 10.3 Å and multiplied by the same factor of 220 for visual clarity.

### Calculation of occupation of the excited states

To simulate the population of excited states in Fig. 3c, the sodium cluster was excited with an exemplary Gaussian pulse

$${E}_{z}\left(t\right)={E}_{0}\exp \left[-\frac{{\left(t-{t}_{0}\right)}^{2}}{2{\left(\Delta t\right)}^{2}}\right]\cos ({2\uppi \nu }_{0}t)$$

with a field strength ${E}_{0}=$ 2.16 V nm^−1^, a centre frequency ${\nu }_{0}=$ 241.8 THz (1 eV), a pulse duration of Δt= 4.4 fs and an offset of ${t}_{0}=$ 12.5 fs. The resulting wavefunction was projected onto the ground state orbitals to retrieve the energy-resolved electronic distribution.

## Online content

Any methods, additional references, Nature Portfolio reporting summaries, source data, extended data, supplementary information, acknowledgements, peer review information; details of author contributions and competing interests; and statements of data and code availability are available at 10.1038/s41566-026-01932-0.

## Supplementary information

Supplementary Video 1 The supplementary video provides the full real-time evolution of the simulated relative charge density Δ*ρ*(*t*) = *ρ*(*t*) – *ρ*_0_ within the tunnel junction (tip–sample distance 16 Å). It shows the continuous temporal progression corresponding to the selected snapshots presented in Fig. 3. The video captures the emergence of geometry-following excitations, the subsequent shake-up dynamics of Δ*ρ*(*t*) and the rapid charge redistribution within the tunnelling gap.

## Source data

Source Data Fig. 1 All data of Fig. 1.

Source Data Fig. 2 All data of Fig. 2.

Source Data Fig. 3 All data of Fig. 3.

Source Data Fig. 4 All data of Fig. 4.

Source Data Extended Data Fig. 1 All data of Extended Data Fig. 1.

Source Data Extended Data Fig. 2 All data of Extended Data Fig. 2.

Source Data Extended Data Fig. 3 All data of Extended Data Fig. 3.

Source Data Extended Data Fig./ 4 All data of Extended Data Fig. 4.

Source Data Extended Data Fig. 5 All data of Extended Data Fig. 5.

Source Data Extended Data Fig. 6 All data of Extended Data Fig. 6.

## Data Availability

The datasets generated and/or analysed during the current study are attached. Any additional data are available from the corresponding authors. Source data are provided with this paper.

## Code availability

All the TD-DFT calculations have been done using the open-source code octopus (https://www.octopus-code.org/documentation/16/).
